# An inexpensive method of small paraffin tissue microarrays using mechanical pencil tips

**DOI:** 10.1186/1746-1596-6-117

**Published:** 2011-12-01

**Authors:** Abdelhadi M Shebl, Khaled R Zalata, Maha M Amin, Amira K El-Hawary

**Affiliations:** 1 Pathology department, faculty of medicine, Mansoura University, Egypt

**Keywords:** Paraffin tissue, microarrays-mechanical, pencil tips

## Abstract

**Background:**

Tissue microarray technology has provided a high throughput means of evaluating potential biomarkers in archival pathological specimens. This study was carried out in order to produce tissue microarray blocks using mechanical pencil tips without high cost.

**Method:**

Conventional mechanical pencil tips (Rotring Tikky II Mechanical Pencil 1.0 mm) were used to cut out 1 mm wax cylinders from the recipient block, creating from 36 to 72 holes. Three cores of tumor areas were punched out manually by using the mechanical pencil tips from donor paraffin embedded tissue blocks and transferred to the holes of the paraffin tissue microarrays.

**Results:**

This technique was easy and caused little damage to the donor blocks. We successfully performed H&E slides and immunodetection without substantial tissue cylinder loss.

**Conclusion:**

Our mechanical pencil tip technique is the most inexpensive easy technique among the literature. It also takes a reasonable amount of time and reduces antibody consumption during immunohistochemistry

## Introduction

Tissue microarray (TMA) technology has provided a high throughput means of evaluating potential biomarkers and therapeutic targets in archival pathological specimens. TMAs facilitate the rapid assessment of molecular alterations in hundreds of different tumors on a single slide [1]. The conventional construction of a TMA block involves the use of a commercial TMA builder instrument to punch the cores from donor blocks, and the transference of these tissue cores to a recipient block, producing blocks with even 1000 tissue cores [2]. There are two types of TMA technique, automated and manual. In automated method one can mark, edit and save punch coordinates using an on-screen display and software tools, while performing visual selection during punching, using magnifying glass or a stereomicroscope as a guide [3]. However, this technique has been restricted to institutions with the funds to buy a tissue puncher/arrayer (from Beecher Instruments, Sun Prairie, WI; cost, at least $7,000) or to let commercial companies do the expensive array construction (custom-built paraffin tissue microarrays with 96 holes, about $900 [MaxArray System], Zymed Laboratories) [4]**^.^**

Manual Tissue Arrayers are commercially available [3]. One can construct a tissue array block in minutes, simply by punching the donor tissue cores using punch needles with plunger and insert to the pre-made paraffin recipient block without the need of specialized equipment. In spite of being handy, portable, simple, and easy to use, its cost ranges from $ 265 for two punch needles with plungers and one - pre-made paraffin recipient block (2 mm × 40 cores) to $4000 for full set manual tissue microarray [5]. Pires et al [2] described a new technique which is based on the construction of TMA needles modifying conventional hypodermic needles to punch tissue cores from donor blocks. They built TMA blocks with more than 300 tissue cores with initial cost of near $ 100.00. In Egypt, we considered $100.00, which equal nearly 600 Egyptian pounds, high cost for paraffin tissue microarrays (PTMAs). So, this study was carried out in order to produce TMA blocks using mechanical pencil tips without the high cost mentioned above.

## Methodology

### Paraffin Tissue Punches

**Figure 1:**
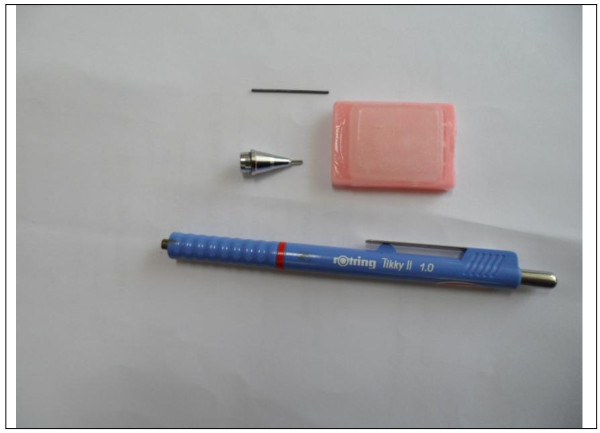
**Mechanical pencil 1.0 mm used to manufacture the paraffin tissue punches**.

Conventional mechanical Pencil tips (Rotring Tikky II Mechanical Pencil 1.0 mm) were used to manufacture the paraffin tissue punches (Figure 1).

### Construction of Recipient Blocks

**Figure 2:**
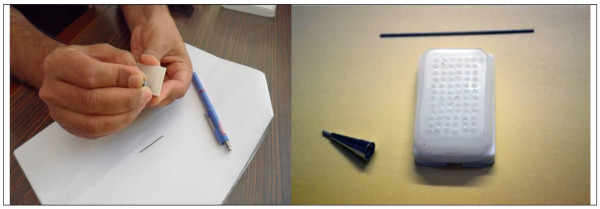
**Mechanical pencil tip 1 mm thick used to cut out 1 mm wax cylinders from the recipient block, creating 72 holes**.

Empty paraffin blocks (recipient blocks) were prepared with standard mould placed on the standard holder. To avoid air bubbles to be trapped under the plastic, the cassettes were warmed to 62°C before filling. After pouring, the paraffin blocks were cooled at room temperature to avoid cracks. Before drilling, the recipient paraffin blocks were examined for air bubbles and paraffin cracks. Mechanical pencil tip 1 mm thick was used to punch out 1 mm wax cylinders from the recipient block, creating from 36 to 72 holes (Figure 2)

### Filling the recipient block

**Figure 3:**
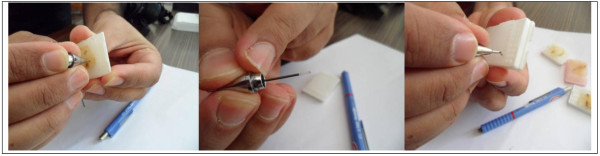
**Mechanical pencil tip used to punch out tumor areas from donor paraffin embedded tissue block and pressed out of the needle gently into the hole with mechanical pencil lead and transferred to the holes of the recipient block**.

Areas containing the most characteristic features of pathologic process on hematoxylin eosin (H&E) stained slides were identified and marked. After putting the marked slides over the surface of paraffin block, areas on the block corresponding to regions on the slide could be identified and marked. We used tissues derived from meningioma for array construction. Tissues were obtained from the histopathological archives of the pathology departments, Mansoura University. Three cores of morphologically representative, non-necrotic tumor areas were cut out manually by using the mechanical pencil tips from donor paraffin embedded tissue blocks. Tissue cores were pressed out of the needle gently with mechanical pencil lead and transferred to the holes of the PTMAs (Figure 3). If a short core was submerged in the PTMA, a second or even a third core from the same donor block could be injected in the same hole. The design of each block was detailed in a TMA map, indicating the position and identification of each core.

Once all cores were attached to the recipient paraffin blocks, melted paraffin was gently poured into the blocks to create and improve the adherence between tissue cores and the recipient block. The PTMAs was incubated on the oven at 60°C for 15 minutes.

### Cutting and Staining

**Figure 4:**
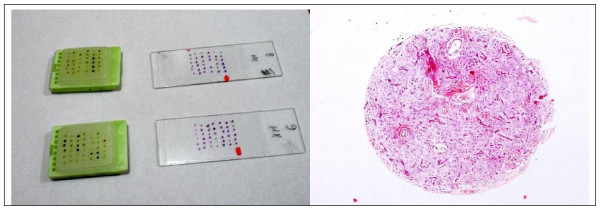
**Macroscopic picture of TMA blocks and H&E stained slides of two tissue microarrays**. Microscopic picture of H&E stained slide showing the whorled architecture of meningioma.

PTMAs blocks were cut with microtome to obtain sections 4-5 μm thick, which were then mounted onto appropriately electrostatic coated microscope slides SuperFrost +, incubated overnight at 40°C and stained with routine H&E (Figure 4).

Immunohistochemical analysis was performed using antibodies against monoclonalmouse anti-human progesterone receptor (Clone PgR636, IR06861, ready to use, Dako, USA), rabbit monoclonal anti-human Ki67 (Clone SP6, RM-9106-R7, ready to use, Neomarkers, Fremont, CA, USA) and rabbit monoclonal anti-human cyclin D1 (Clone SP4, RM-9104-R7, ready to use, Neomarkers, Fremont, CA, USA), according to the manufacturer's instructions. Antigen retrieval was done using EDTA retrieval solution PH 9 in a standard microwave oven for 10 minutes.

### Microphotography

All H&E and immunohistochemistry images were analyzed using a Nikon Eclipse 600 microscope (Nikon, Burlingame, CA). Images were photographed with a Nikon DXM 1200 digital camera

## Results

**Figure 5:**
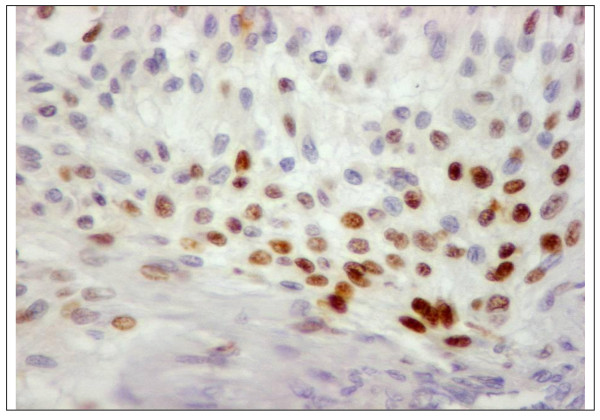
**Nuclear staining of progesterone receptors of one core of multiple tumor arrays of meningioma (DAB, × 400)**.

We constructed 17 TMA blocks using this mechanical pencil tip technique. The time of construction of these blocks was 1 hour to make one 72-cores block. This technique was also easy (once the exact areas are marked on H&E slides, one can punch the donor blocks and construct the TMA block following the designed map). There was little damage to the donor blocks from the punches. There were little core losses (about 2%), most of them due to consumption of one or more tissue cores during microtomy, rarely to section falling off from slides during technical procedures. We successfully performed immunodetection without substantial tissue cylinder loss during antigen retrieval or washes, 65.6% of our cases of meningioma show presence of progesterone receptors (Figure 5).

## Discussion

TMA technology is a great improvement over conventional procedures of doing tests like immunohistochemistry, FISH and mRNA ISH on each tissue sample separately. One of the major drawbacks of TMA technology is the high cost of TMA facilities. The high cost of the array machines limits its use in general practice in many countries [6]. Efforts have been made by many researchers to devise more cost effective TMA construction techniques [2,7-11]. Some of those researchers used bone marrow trephine biopsy needles or other types of needles, however our mechanical pencil tip method have proven to be much more cheap and easy. The cost of our simple method is < 5 $ USD for 1 mm × 72 core paraffin arrays. We choose our mechanical pen of size 1 mm. The standard array needles come in 0.6, 1.0, 1.5, 2.0 mm diameter [11]. Many workers consider the small 0.6 mm cores as the standard of practice. Use of smaller core diameters allows for a greater number of cores to be extracted from the lesion and a greater number of cores that can fit into the TMA block. In addition, they tend to inflict little damage on the donor and recipient blocks. The larger core sizes have the advantages of being more robust and the cores are more difficult to damage during handling. However, these larger sizes can lead to increased likelihood of difficulty in extracting the cores from the blocks as well as greater chance of the blocks being broken or cracked during the TMA generation process [12]. We found that 1 mm diameter of the tissue cylinders maintained recognizable histological features of the arrayed tissues, and offered more tissue surface to evaluate immunostaining. This cylinder size also makes sampling of lesions easier, more accurate and can reduce sectioning artifacts such as the splitting of sections on the hot water bath.

Even though the arrays that we described contain a small number of samples compared with other tissue microarrays, they are still a good alternative in many laboratories in developing countries. This technique can be used to construct microarrays for use in research where more than 12 different tissues/tumors in one section can be stained together. We used 36 to 72 cores of 1 mm thick. Using this number of cores can accommodate from 12 to 24 (three cores per case) cases in one block with a cost < 5 $ USD. Because it is easy to modify the number of cores in the array, it is easy to adapt this to individual labs and requirements. Vogel, [13] described a method that allows the construction of PTMAs with tissue cores only 0.43 mm in diameter in order to conserve the minute quantities of available tumor tissue and to store the greatest possible number of tissue cores in one block. So, we will try to construct PTMAs by the use of mechanical pencil tips with diameter 0.5 mm. By reducing the diameter of the tissue cores, we can achieve a higher density of specimens in a PTMA. This density might also be increased by reducing the distance between the tissue core biopsies.

We successfully performed immunodetection of progesterone receptors in tissue microarrays of meningioma. The staining patterns were in agreement with published data as 65.6% of our cases show presence of progesterone receptors. This is in concordance with results of Pravdenkova et al. [14] who has documented the presence of progesterone receptors in 48 to 88% of cases of meningiomas.

To conclude, our mechanical pencil tip technique is the most inexpensive easy technique among the literature. It also takes a reasonable amount of time and reduces antibody consumption. The availability of this economic TMA will enable every researcher to perform studies involving thousands of samples rapidly. Therefore, our method of TMAs may lead to a significant improvement in basic research especially in developing countries.

## Competing interests

The authors declare that they have no competing interests. The authors don't have any financial interests with any of the commercial products mentioned in this article.

## Authors' contributions

This paper was carried out in collaboration between all authors. AMS and KRZ defined the research idea. All cases were examined and diagnosed by KRZ and AHS. MMA and AKE collected and analyzed the data. AKE wrote the paper. All authors read and approved the final manuscript.
